# Why tocilizumab could be an effective treatment for severe COVID-19?

**DOI:** 10.1186/s12967-020-02339-3

**Published:** 2020-04-14

**Authors:** Binqing Fu, Xiaoling Xu, Haiming Wei

**Affiliations:** 1 Institute of Immunology and the CAS Key Laboratory of Innate Immunity and Chronic Disease, School of Life Science and Medical Center, University of Science and Technology of China, Hefei, Anhui 230001 China; 2 Hefei National Laboratory for Physical Sciences at Microscale, University of Science and Technology of China, Hefei, Anhui 230001 China; 3 The First Affiliated Hospital of USTC, Division of Life Sciences and Medicine, University of Science and Technology of China, Hefei, Anhui 230001 China

**Keywords:** COVID-19, SARS-CoV-2, Acute respiratory distress syndrome (ARDS), Inflammatory storm, IL-6, Tocilizumab

## Abstract

A severe pneumonia-associated respiratory syndrome caused by a new coronavirus was identified in December 2019 (COVID-19), spread rapidly and has become a world-wide public health challenge. About 25% of COVID-19 patients experienced severe complications including acute respiratory distress syndrome (ARDS), and even progressed into an intensive care unit (ICU) admission and died. The exploration for the mortality causes and advancing novel therapeutic development of severe COVID-19 is crucial at the moment. The biopsy samples analysis at autopsy suggested that increased alveolar exudate caused by aberrant host immune response and inflammatory cytokine storm probably impedes alveolar gas exchange and contributes to the high mortality of severe COVID-19 patients. Our research has identified that pathogenic T cells and inflammatory monocytes incite inflammatory storm with large amount of interleukin 6, therefore monoclonal antibody that targets the IL-6 pathways may potentially curb inflammatory storm. Moreover, Tocilizumab treatment that blocking IL-6 receptors showed inspiring clinical results including temperature returned to normal quickly and respiratory function improved. Therefore, we suggest that Tocilizumab is an effective treatment in severe patients of COVID-19 to calm the inflammatory storm and reduce mortality.

## Introduction

In the past decades, two known pathogenic human coronaviruses, severe acute respiratory syndrome CoV (SARS-CoV) and Middle East respiratory syndrome CoV (MERS-CoV), have been reported to damage the respiratory tract and cause high morbidity and mortality [1]. Severe acute respiratory syndrome coronavirus 2 (SARS-CoV-2) is a newly discovered coronavirus, was reported at December 2019 (2019-nCoV) in the city Wuhan, Hubei province, China [2]. Up to 21th of March 2020, 81,416 cases have been reported with 3261 fatal cases according to the Chinese Center for Disease Control and Prevention (CDC). Meanwhile, 190,000 cases have been reported with 7992 fatal cases in other countries except China. In Italy, to date there are about 47.021 infected and 4.032 deaths [3]. A global outbreak of the SARS-CoV-2 caused Corona Virus Disease (COVID-19) seems inevitable. Among these COVID-19 patients, most of them have the common symptoms including fever, cough, and myalgia or fatigue at onset. The majority of patients can recover, however, about 25% of patients will progress into severe complications including acute respiratory distress syndrome (ARDS), which may worsen rapidly into respiratory failure, need an intensive care unit (ICU) and even cause multiple organ failure [4, 5]. Therefore, the exploration for the mortality causes and advancing novel therapeutic development of severe COVID-19 is crucially important at the moment.

## What is the crucial cause for mortality in COVID-19?

Although virus-induced cytopathic effects and viral evasion of host immune responses are believed to be important in disease severity, studies from humans who died of SARS and MERS suggested that an aberrant host immune response resulting in an inflammatory cytokine storm and lethal disease [1]. Similar to the inflammatory cytokines in SARS and MERS, patients with COVID-19 also have increased plasma concentrations of inflammatory cytokines, such as tumour necrosis factor α (TNF-α),interleukins (IL) 2, 7, and 10, granulocyte-colony stimulating factor (G-CSF), monocyte chemoattractant protein 1, macrophage inflammatory protein 1 alpha, and interferon-γ-inducible protein 10, especially in ICU patients, which implied a cytokine storm occurred [4].

Moreover, COVID-19 patients have decreased lymphocytes in peripheral blood and characteristic pulmonary ground glass changes on imaging [4, 5]. Most importantly, in the biopsy samples at autopsy from patients who died from COVID-19, histological examination showed bilateral diffuse alveolar damage including edema, proteinaceous exudate, focal reactive hyperplasia of pneumocytes with patchy inflammatory cellular infiltration, and multinucleated giant cells [6, 7]. It also has been recovered from autopsy examination that Type II alveolar epithelial cells proliferate markedly, with some cells exfoliated. The alveolar septum is hyperemic, edematous, with clear intravascular thrombosis. Focal monocytes, lymphocytes and plasma cells are infiltrating into pulmonary interstitium. Immunohistochemistry results showed positive for immunity cells including CD3, CD4, CD8, CD20, CD79a, CD5, CD38 and CD68 [8]. These phenomena further suggest severe pulmonary inflammatory immune cells exist in SARS-CoV-2 infection. Therefore, increased alveolar exudate caused by aberrant host immune response and inflammatory cytokine storm probably impedes alveolar gas exchange and contributes to the high mortality of severe COVID-19 patients.

## IL-6 is a potential blocking target to calm inflammatory storm

**Fig. 1:**
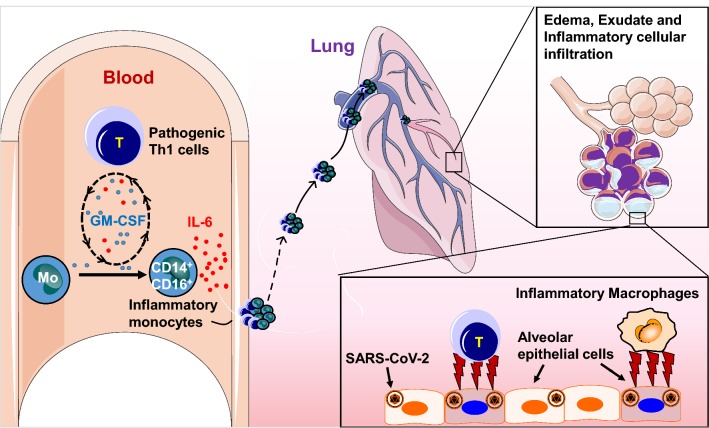
Pathogenic T cells and inflammatory monocytes with high IL-6 secretion may enter the pulmonary circulation in large numbers,incite the inflammatory storm and lead an immune disorder in severe COVID-19 patients

Inflammatory storm refers to an excessive inflammatory response flaring out of control and the immune system gone awry. To identify which kind of immune cells are involved in and which inflammatory cytokine is the critical target in these severe COVID-19 patients, we analyzed peripheral blood samples from patients with severe or critical COVID-19 from The First Affiliated Hospital of University of Science and Technology of China and observed monocytes and T cells from severe or critical COVID-19 patients decreased significantly compared to normal controls. These aberrant pathogenic T cells from critical ICU care COVID-19 patients showed activated characteristic accompanied with co-expressing IFN-γ and GM-CSF. This phenomenon aroused our alarm, for GM-CSF has the capability to control diverse pathogenic capabilities of inflammatory myeloid cells, especially monocytes [9]. As expected, inflammatory monocyte with CD14^+^CD16^+^ phenotype exists in peripheral blood of COVID-19 patients and has larger population in critical COVID-19 patients from ICU. Note that without any re-stimulation with PMA or incubation with monensin, large amount of IL-6 could be tested from these inflammatory monocytes especially in ICU patients. Therefore, these pathogenic Th1 cells (GM-CSF^+^IFN-γ^+^) and inflammatory monocytes (CD14^+^CD16^+^ with high expression of IL-6) exist especially in critical ICU COVID-19 patients [10]. Given that large amount of mononuclear inflammatory lymphocytes have been observed in the biopsy samples at autopsy from COVID-19 patients, we believe that these pathogenic T cells and inflammatory monocytes may enter the pulmonary circulation in large numbers and incite inflammatory storm in severe or critical COVID-19 patients (Fig. 1).

## Tocilizumab treatment is effective to reduce the mortality of severe COVID-19

**Fig. 2:**
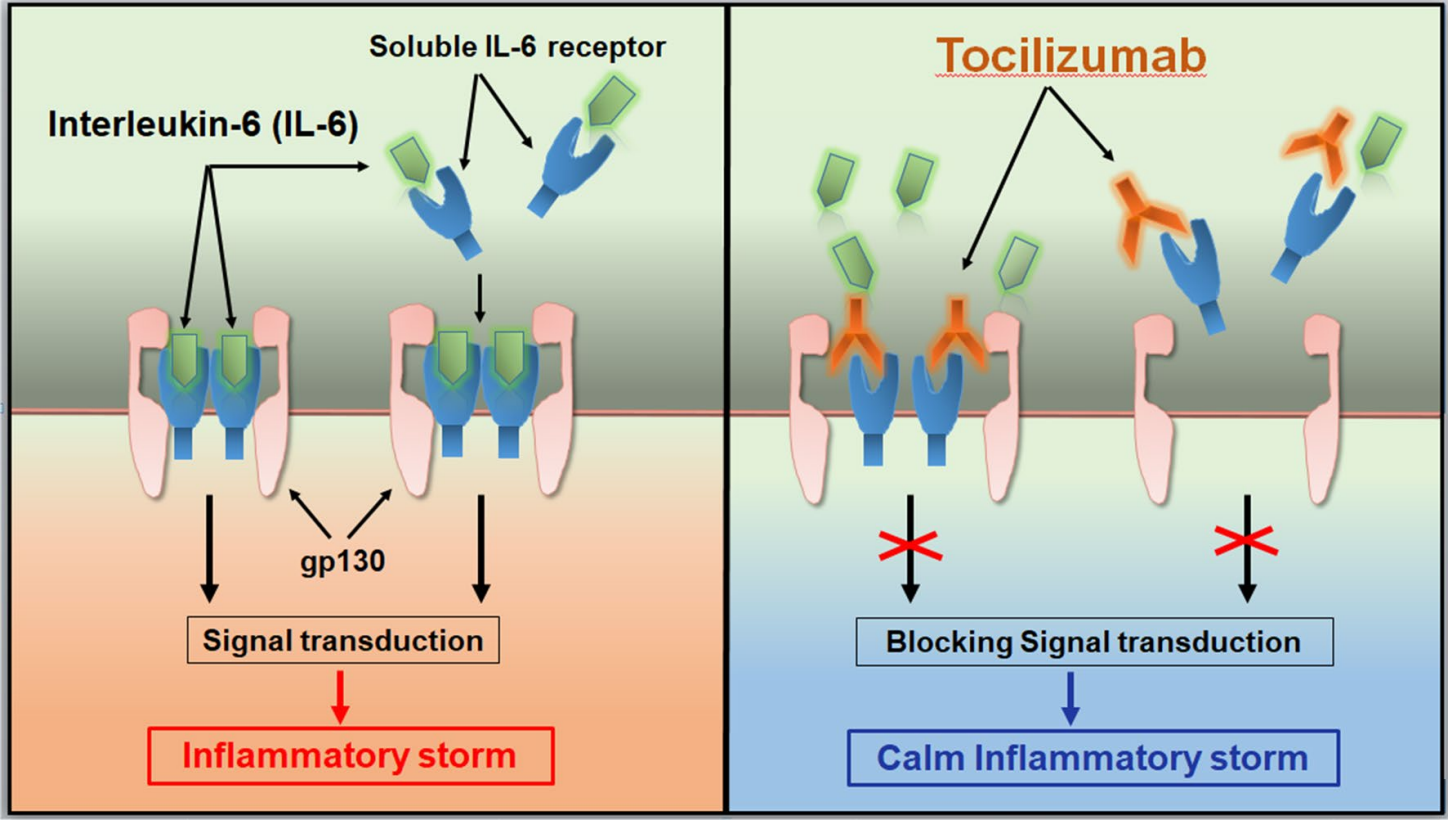
Tocilizumab calms the inflammatory storm through blocking IL-6 receptors

Tocilizumab is the first marketed IL-6 blocking antibody through targeting IL-6 receptors and has proved its safety and effectiveness in therapy for rheumatoid arthritis (Fig. 2). In order to verify whether targeted IL-6, may potentially be the effective and safe way to reduce mortality of COVID-19, 21 patients diagnosed as severe or critical COVID-19 from The First Affiliated Hospital of University of Science and Technology of China and Anhui Fuyang Second People’s Hospital were recruited and given tocilizumab therapy (Table 1). Patients received standard treatment according to the Diagnosis and Treatment Protocol for COVID-19 (7^th^ edition), including lopinavir, methylprednisolone, other symptom relievers and oxygen therapy. The results of tocilizumab treatment are inspiring. The temperature of all the patients returned to normal very quickly. The respiratory function and all other symptoms improved remarkably. Among these 21 patients, 20 patients have been recovered and discharged within 2 weeks after the tocilizumab therapy. One left patient is recovering and out of ICU care. No adverse drug reactions were reported during the treatment with tocilizumab [11]. With these promising preliminary clinical results, we further launched the multicenter, large-scale clinical trials (ChiCTR2000029765) and have already about 500 severe or critical patients treated this way.

**Table 1:** Patients can be considered to use Tocilizumab and the exclusion criteria

Patients can be considered to use Tocilizumab	Exclusion criteria
(1) Patients diagnosed with COVID-19 with high risk factors, severe or critical patients	(1) Patients who are participating in clinical trials of other drugs
(2) Increased concentration of IL-6 levels	(2) Pregnant or lactating women
(3) The patient or authorized family member agrees to use Tocilizumab treatment and sign the informed consent	(3) Rheumatoid immune-related diseases
	(4) Long-term oral medication of anti-rejection drugs or immunoregulatory drugs
	(5) Hypersensitive to Tocilizumab or any pharmaceutical excipients
	(6) Active pulmonary tuberculosis patients with bacterial and fungal infections
	(7) Organ transplant patients
	(8) Patients with mental disorders
	(9) ALT/AST > 5ULN, neutrophil < 0.5 × 10^9^/L, platelet < 50 × 10^9^/L

The immunotherapy strategy about Tocilizumab treatment has been formally included in the diagnosis and treatment program of COVID-19 (7th edition) of the national health commission of China since 3th March 2020 as following: Tocilizumab can be used in patients with extensive bilateral lung lesions opacity or in severe or critical patients, who have elevated laboratory detected IL-6 levels. The first dose is 4–8 mg/kg (the recommended dose is 400 mg, diluted to 100 ml with 0.9% normal saline, and the infusion time is more than 1 h). For patients with poor initial efficacy, an additional application can be made after 12 h (the dose is the same as before). The maximum number of times of administration is two, and the maximum dose of a single dose should not exceed 800 mg. Note that patients with allergic reactions, such as tuberculosis and other active infection are contraindicated. We suggest that IL-6 concentrations can be detected if fever persists for more than 3 days. By chemiluminescence detection, if serum IL-6 content is over 20 pg/ml, Tocilizumab can be used. The IL-6 will be temporarily increased in serum in the next few days, for its receptors have been blocked by Tocilizumab. Together, Tocilizumab treatment is recommended to reduce the mortality of severe COVID-19.

## Discussion

All three coronaviruses, including SARS-CoV, MERS-CoV and SARS-CoV-2, induce aberrant non-effective host immune responses that are associated with severe lung pathology. The new SARS-CoV-2 additionally causes serious alveolar mucus infiltration and multiple organ failure. As the SARS-CoV-2 continues to spread, the numbers of fatal cases rise exponentially in many countries, advancing novel therapeutic development becomes crucial to minimize the number of deaths from COVID-19. In the absence of specific antiviral drugs, existing host-directed therapies could potentially be repurposed to treat COVID-19. China’s plan of Tocilizumab treatment has shown its remarkable effectiveness and safety in clinical practice over the past 2 months, hoping it will benefit other countries fighting the pandemic and reduce the mortality of severe COVID-19 as well.

## Data Availability

Not applicable.

## Abbreviations

SARS-CoV: Severe acute respiratory syndrome CoV
MERS-CoV: Middle east respiratory syndrome CoV
SARS-CoV-2: severe acute respiratory syndrome coronavirus 2 outbreak in 2019
COVID-19: The SARS-CoV-2 caused Corona Virus Disease
GM-CSF: Granulocyte-macrophage colony-stimulating factor
IL-6: Interleukin-6
ARDS: Acute respiratory distress syndrome
ICU: Intensive care unit
